# Supplementary material for: DMRforPairs: identifying Differentially Methylated Regions between unique samples using array based methylation profiles
Source: BMC Bioinformatics. 2014 May 15;15:141. doi: 10.1186/1471-2105-15-141 (PMC4046028; doi:10.1186/1471-2105-15-141)
Supplement: Additional file 2 — DMRforPairs output for the comparison of A431-MCF7 and NA17018-NA17105. Please start from the HTML files in each folder. Available via the BMC Bioinformatics website. [file 1471-2105-15-141-S2.zip › 1394847754114233_MOESM2_ESM/A431_MCF7/figures/17950.pdf]

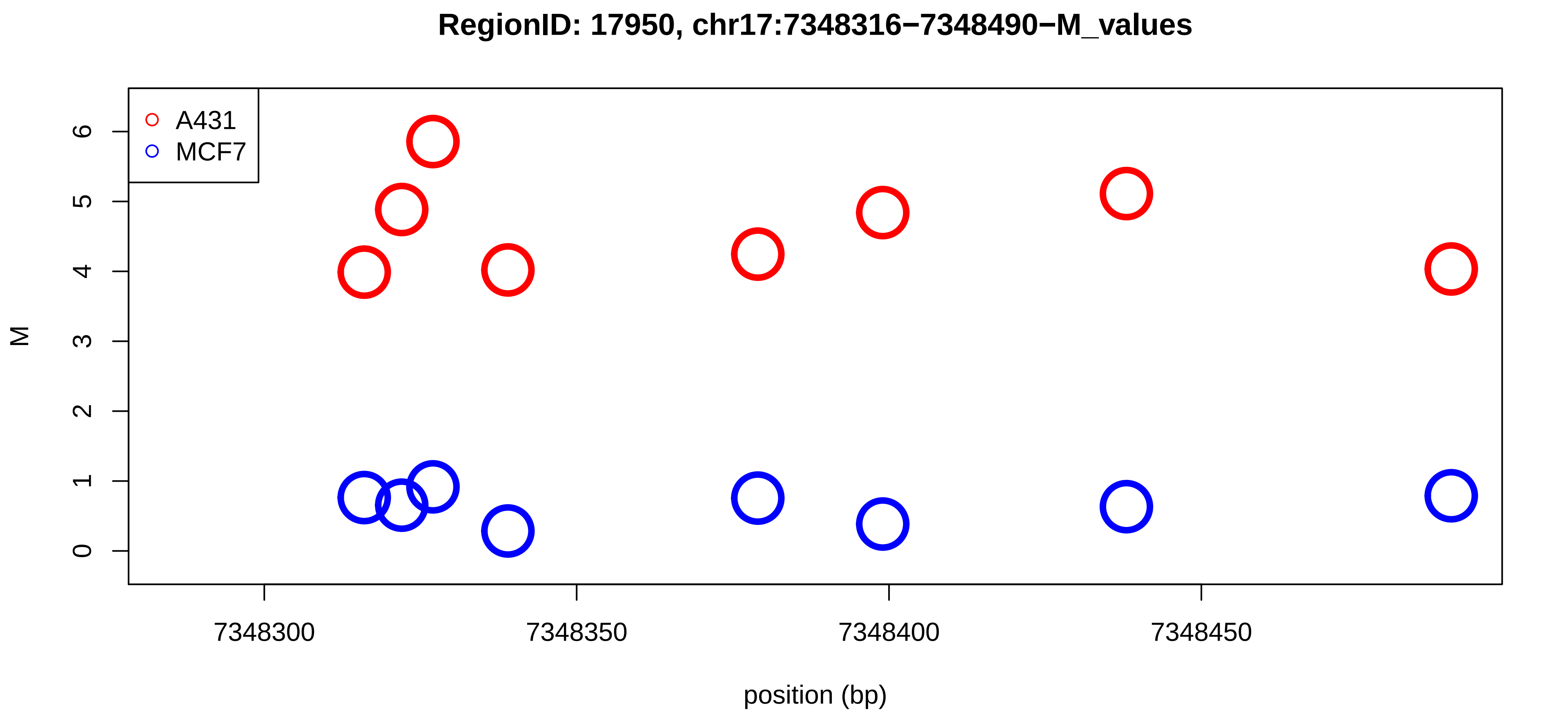
RegionID: 17950, chr17:7348316−7348490−M_values
A431
MCF7
M
0
1
2
3
4
5
6
7348300
7348350
7348400
7348450
position (bp)

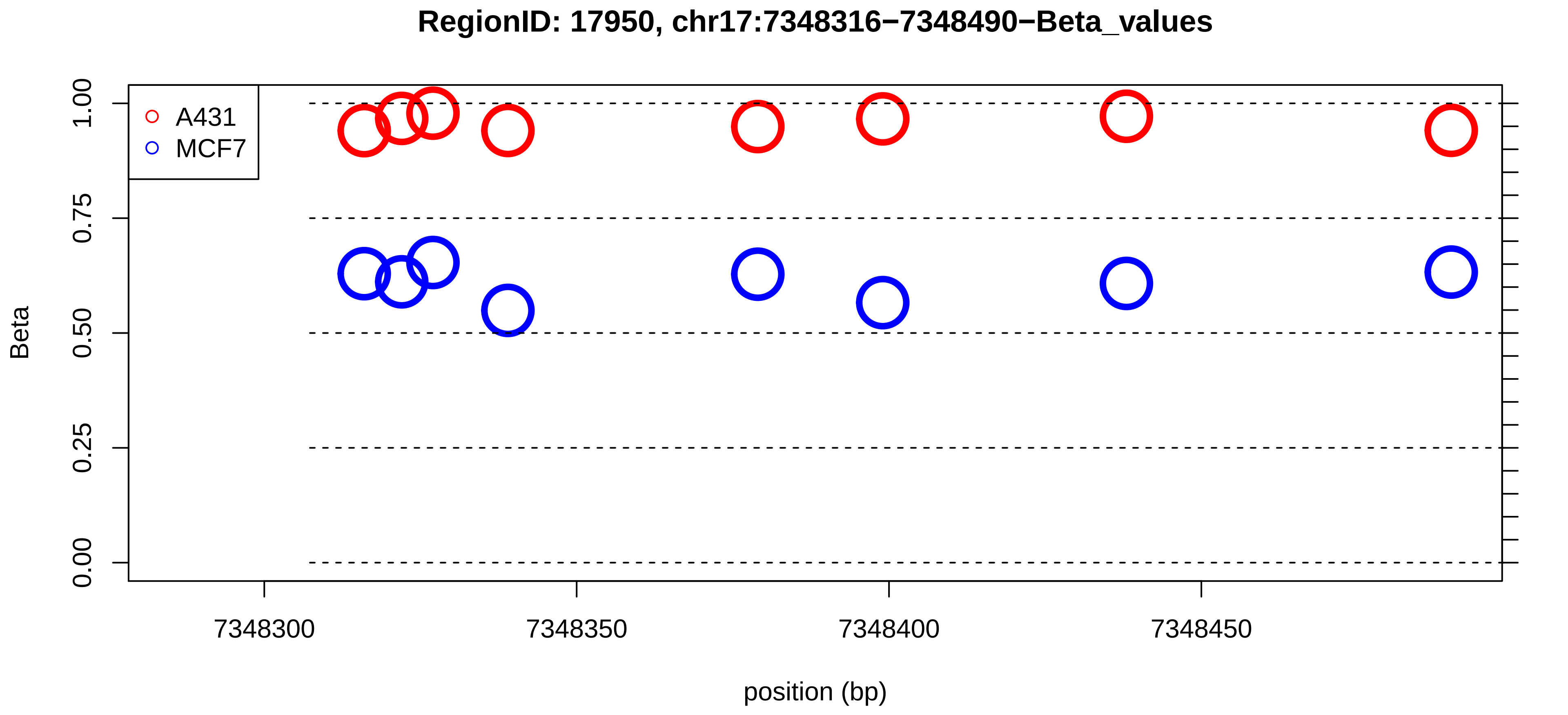
RegionID: 17950, chr17:7348316−7348490−Beta_values
A431
MCF7
Beta
0.00
0.25
0.50
0.75
1.00
7348300
7348350
7348400
7348450
position (bp)

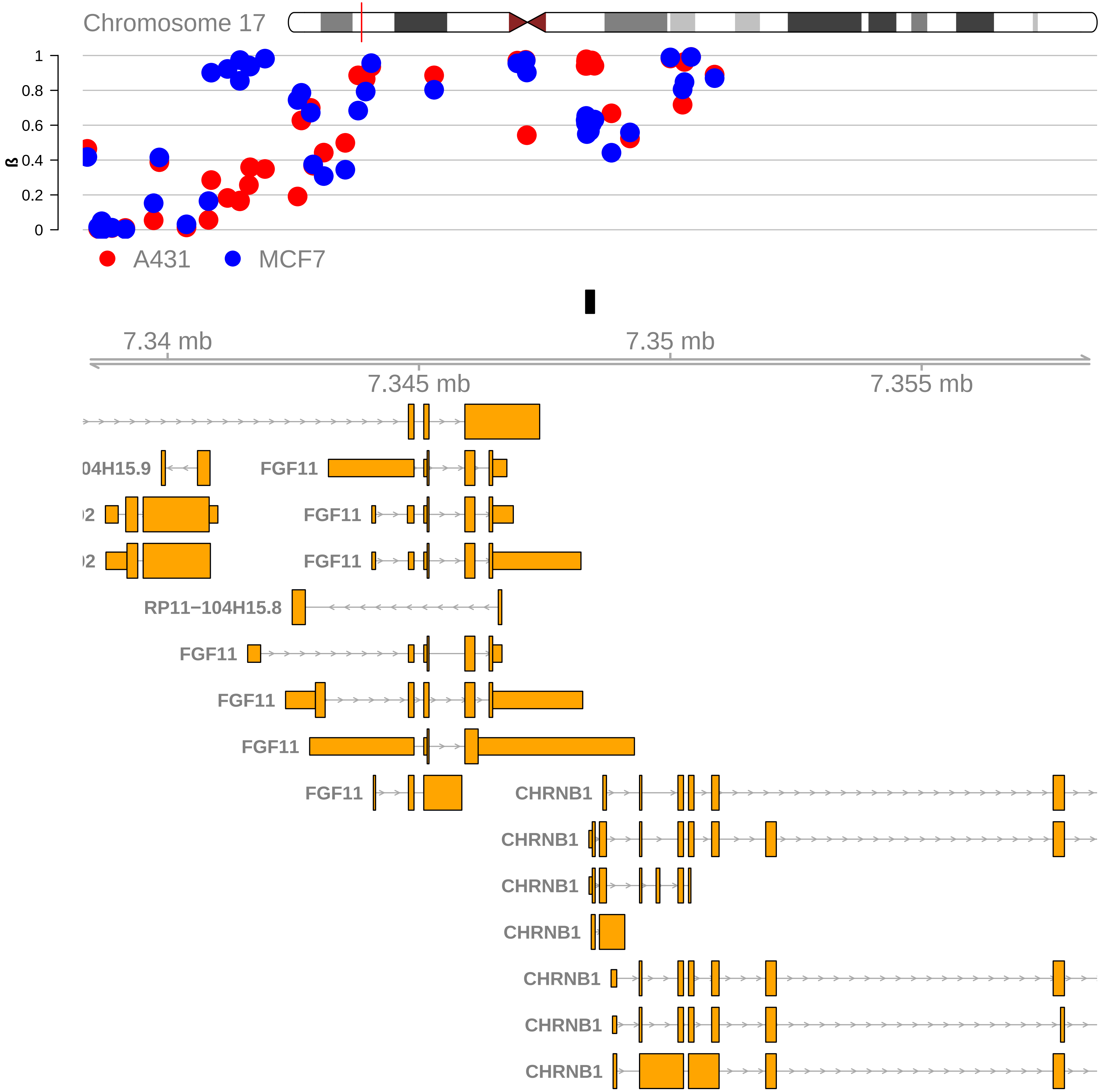

Chromosome 17
1
0.8
0.6
ß 0.4
0.2
0
A431
MCF7
7.34 mb
7.345 mb
7.35 mb
7.355 mb
04H15.9
FGF11
)2
FGF11
)2
FGF11
RP11−104H15.8
FGF11
FGF11
FGF11
FGF11
CHRNB1
CHRNB1
CHRNB1
CHRNB1
CHRNB1
CHRNB1
CHRNB1
